# A Mechanical Device for Illustrating the Movement of the Lung in Penetrating Wounds of the Chest

**Published:** 1894-07

**Authors:** 


A MECHANICAL DEVICE FOR ILLUSTRATING THE MOVEMENTS OF THE LUNG IN PENETRATING WOUNDS OF THE CHEST.—Dr. Andrew H. Smith, of New York City, showed before the American Climatological Association an apparatus which consists of two bellows, operated by a handle common to both, representing the thoracic cavities, and each containing an elastic bag representing the lung. The top of each bellows is of glass. A slot on each side, covered by a slide, represents a wound of dimensions variable at pleasure. Tubes representing the bronchi and trachea connect the two bags. With the slot of one side wide open and the bag on that side disconnected from its fellow, it is seen that the movements of the bellows are without effect upon the bag. But when the connection is re-established, it is evident that the bag receives air from its fellow when the handle is depressed, and that it collapses when the handle is lifted, its movements being exactly the reverse of those of the bag on the other side. When the device representing the glottis is partly closed, this reverse movement is very marked.—*International Medical Magazine.*

